# Influence of response shift and disposition on patient-reported outcomes may lead to suboptimal medical decisions: a medical ethics perspective

**DOI:** 10.1186/s12910-019-0397-3

**Published:** 2019-09-11

**Authors:** Iris D. Hartog, Dick L. Willems, Wilbert B. van den Hout, Michael Scherer-Rath, Tom H. Oreel, José P. S. Henriques, Pythia T. Nieuwkerk, Hanneke W. M. van Laarhoven, Mirjam A. G. Sprangers

**Affiliations:** 1 Department of Medical Psychology, Amsterdam UMC, University of Amsterdam, Meibergdreef 9, 1105 AZ Amsterdam, The Netherlands; 2 Faculty of Philosophy, Theology and Religious Studies, Radboud University Nijmegen, Postbus 9103, 6500 HD Nijmegen, The Netherlands; 3 Department of Medical Ethics, Amsterdam UMC, University of Amsterdam, Meibergdreef 9, 1105 AZ Amsterdam, The Netherlands; 4 Department of Medical Decision Making & Quality of Care, Leiden University Medical Center, Postbus 9600, 2300 RC Leiden, The Netherlands; 5 Department of Cardiology, Amsterdam UMC, University of Amsterdam, Meibergdreef 9, 1105 AZ Amsterdam, The Netherlands; 6 Department of Medical Oncology, Cancer Center Amsterdam, Amsterdam UMC, University of Amsterdam, Meibergdreef 9, 1105 AZ Amsterdam, The Netherlands

## Abstract

**Background:**

Patient-reported outcomes (PROs) are frequently used for medical decision making, at the levels of both individual patient care and healthcare policy. Evidence increasingly shows that PROs may be influenced by patients’ response shifts (changes in interpretation) and dispositions (stable characteristics).

**Main text:**

We identify how response shifts and dispositions may influence medical decisions on both the levels of individual patient care and health policy. We provide examples of these influences and analyse the consequences from the perspectives of ethical principles and theories of just distribution.

**Conclusion:**

If influences of response shift and disposition on PROs and consequently medical decision making are not considered, patients may not receive optimal treatment and health insurance packages may include treatments that are not the most effective or cost-effective. We call on healthcare practitioners, researchers, policy makers, health insurers, and other stakeholders to critically reflect on why and how such patient reports are used.

## Background

Medical decisions at the individual patient (micro) level as well as at the healthcare policy (macro) level increasingly involve patients’ self-reports. These patient-reported outcomes (PROs), such as health-related quality of life (HRQoL), can only be provided by patients. For example, pain, fatigue, difficulty performing tasks, satisfaction, and overall quality of life reflect patients’ highly personal experiences. The emergence of PROs is the result of a more patient-centred approach in healthcare and research. Moreover, treatments increasingly yield comparable clinical outcomes such as survival, while PROs may vary widely.

All data reported by patients themselves may be subject to unmeasured influences. We focus here on two types of such influences that have not been given due attention. The first is **response shift**, which is defined as a change in the meaning of one’s self-evaluation, as a result of changes in internal standards, values, and/or conceptualization of the PRO [1]. These shifts are often induced by health-changing events, such as falling seriously ill or undergoing treatment. For example, a patient undergoing chemotherapy that causes severe fatigue may change her internal standard for fatigue severity as a result of adaptation. Consequently, her scores may indicate lower levels of fatigue than would be expected, given the impact of the chemotherapy [2]. Thus, whereas these response shifts are often a sign of adaptation, they may distort the interpretation of changes in PRO scores over time.

The second type of unmeasured influences is **disposition**, referring to stable characteristics that people exhibit across circumstances and time, e.g. personality. There is ample evidence that people have a disposition for certain attributes that influence PROs, e.g. optimism/pessimism, denial/catastrophizing, and feeling happy/unhappy [3]. Patients’ dispositions affect individual self-evaluations and may lead to differences in PRO results among patients with the same health state [4].

In this paper we define ‘health state’ as the level of ‘statistically normal biological functioning’ [5], using the biomedical definition of health as the absence of pathology [6]. The biomedical perspective aims to distinguish people’s health from their own standards and preferences, which may be adaptive and culturally informed [7]. This thus enables us to theoretically distinguish the contribution of people’s health states to the reported HRQoL from the contribution of response shifts and dispositions.

Currently, the potential influence of response shifts and dispositions on medical decision making is only taken into account to a limited extent - and usually only implicitly - in consultation rooms, and not at the level of healthcare policy. Consequently, medical decisions may be taken on insufficient grounds and hence may be suboptimal. At the micro level, patients may not receive optimal treatment, as argued below. At the macro level, basic health insurance packages may include treatments that are not the most clinically effective or cost-effective. At present, it is unknown which decisions may be influenced and in what ways. Therefore, here we aim to identify the possible influences of response shifts and dispositions on PROs that have unintended consequences for medical decision making. To illustrate these influences, we provide hypothetical scenarios at the individual patient and policy level. We analyse these examples from three ethical perspectives for the micro level and two ethical theories of distribution for the macro level, to clarify which consequences are problematic – either because they are detrimental to individual patients or to society as a whole.

## Main text

### Patient-reported outcomes in clinical studies

Clinical studies - including PROs - form the basis of medical decision making, both in the consultation room and on the policy level. Response shift may systematically influence PROs in several types of clinical studies [8]. In cross-sectional studies, response shifts induced by events in the past may result in higher or lower PROs than would be expected based on patients’ health states. During prospective cohort studies, patients may undergo new response shifts that lead to an underestimation or overestimation of health changes over time. Similarly, in randomized controlled trials (RCT) and, consequently, in cost-effectiveness studies, the compared treatments may induce different degrees or directions of response shift in the same or even in different PROs. As a consequence, treatment effects may be underestimated or overestimated. For example, health deterioration due to illness progression or treatment may require adaptation by patients. As a result, a greater response shift may be induced by such a treatment than by treatments resulting in less health deterioration. This is illustrated in the following scenario.

#### Scenario 1

**Fig. 1:**
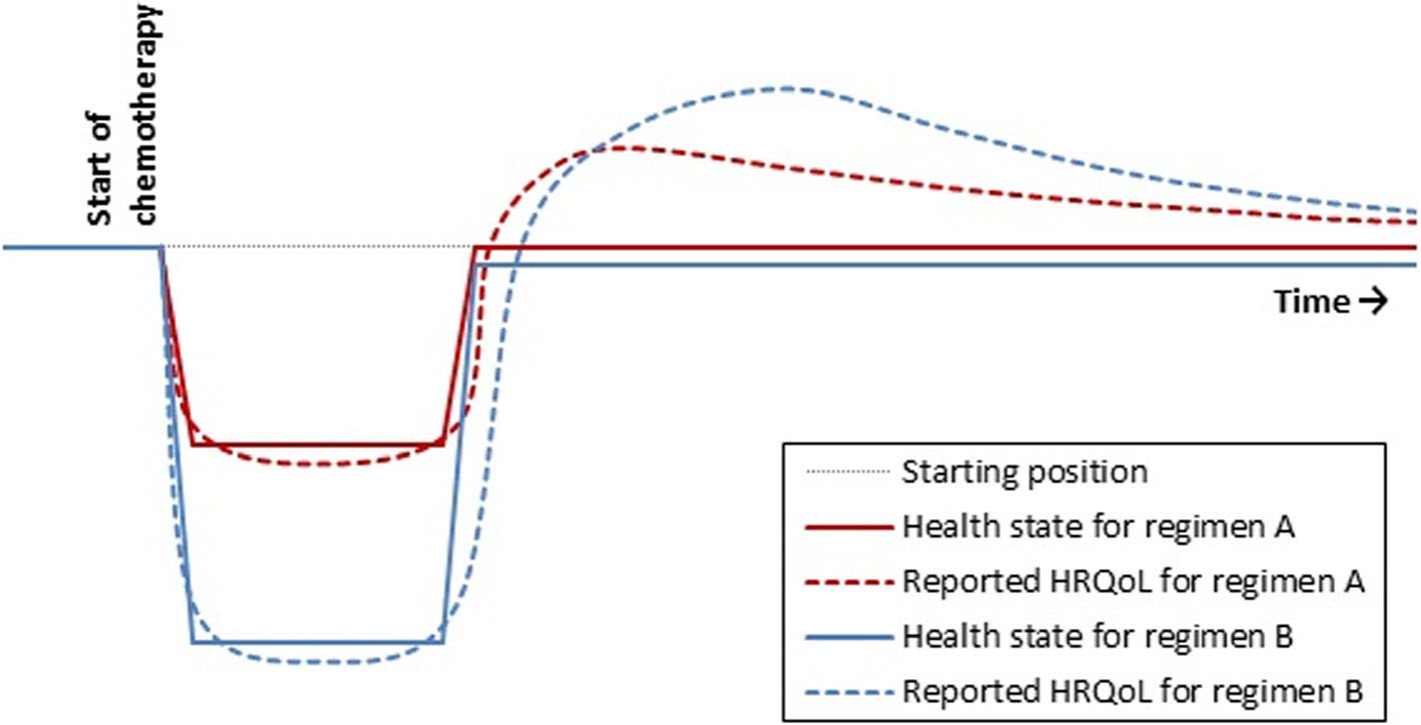
Health states and reported HRQoL after treatment with regimen A versus regimen B (Scenario 1)

An RCT in patients with metastatic gastric cancer is conducted to compare treatment with a doublet of cytotoxic agents followed by a third cytotoxic agent upon progression (regimen A; standard care) with a combination treatment with a triplet of cytotoxic agents (regimen B). The survival outcomes of both treatments turn out to be similar, but at follow-up, health states for group A are slightly better. Moreover, patients in group B experience more acute side effects during treatment, including neutropenic fever. This results in a greater response shift for group B than for group A. At follow-up, these acute side effects have disappeared. The stronger response shift in group B results in higher reported HRQoL scores than in group A, even though their health state is slightly worse, as is shown in Fig. 1.

Disposition may also influence PROs in clinical studies. For example, optimism may lead to higher HRQoL scores than would be expected based on health state, and rigidity may lower the changeability of HRQoL and thus influence conclusions about the effects of treatments. Furthermore, patients with a certain disposition may agree to participate in studies more often than other patients. Dispositions are not likely to influence the results of RCTs and consequently of cost-effectiveness studies, as group differences at baseline are due to chance. However, in cross-sectional or prospective studies, disposition may *systematically* influence PROs if groups of patients have different dispositions that are related to the outcome, i.e. if a disease is associated with a certain disposition. This is illustrated in the following scenario.

#### Scenario 2

A cross-sectional study is conducted to compare HRQoL of two groups of patients with congenital heart disease: pulmonary valve stenosis and Marfan’s syndrome with mitral valve stenosis. Research suggests that psychological dispositions that negatively impact HRQoL may be part of the phenotype of Marfan’s syndrome [9]. This could result in Marfan patients reporting lower levels of HRQoL than patients with pulmonary stenosis, given *the same health state*. As a result, the health state of Marfan patients may be systematically underestimated.

### Medical decision making in the consultation room: ethical principles

As shared decision making (SDM) is becoming more important, patients’ self-evaluations and preferences are increasingly taken into account [10]. In this context, healthcare practitioners may inform patients about published PRO data to support their decision making. Thus, response shifts and dispositions may influence SDM to the extent that it is informed by self-reports. Below, we will discuss different types of influences and provide examples with consequences from an ethical perspective.

#### Three ethical principles

We use three principles for moral reasoning in biomedical ethics that are relevant for the level of individual patient care: nonmaleficence, beneficence and respect for autonomy [11]. The first principle, nonmaleficence, supports avoidance of harm to the patient and is based on the ancient maxim, ‘First, do no harm’. In many cases, this principle is considered together with the principle of beneficence, for example, in weighing the benefits and risks of a certain treatment for a patient. The second principle is beneficence, and refers to acting in the best interest of the patient and promoting goods such as health and wellbeing. This includes relieving, lessening, or preventing harm, such as pain and suffering, disease, disability, and death. The third is respect for autonomy. This principle implies respect for the patient’s capacity for self-determination, i.e. respecting and supporting autonomous decisions of the patient. In medical practice, this means that healthcare practitioners usually present treatment options and make recommendations. Patients, in collaboration with their healthcare practitioners, make (informed) decisions about accepting or refusing treatments, partly based on personal values and beliefs [12].

The fourth principle, i.e. justice, may also be at stake at the level of individual patient care, in the sense of equal treatment among the patient populations of individual healthcare practitioners. However, we did not include this principle as we consider it less relevant for individual patient care. For healthcare practitioners, over- or undertreatment of a patient is problematic as such, and not only in relation to the care provided to other patients. Neither is distributive justice considered relevant. In most Western countries at least, in the consultation room, healthcare practitioners are not concerned with the just allocation of resources in healthcare, but rather with providing good healthcare for each individual patient.

#### Influences of response shift and disposition on SDM

We can distinguish three types of influence of response shift or disposition on SDM. First, they may have influenced published PRO results that are used in the decision-making process (for an example and its ethical analysis, see Table 1). Second, response shifts and dispositions may influence patients’ own self-reports. These self-reports may be provided by questionnaires or symptom diaries, or informally, in response to a physician’s enquiries. Third, response shifts and dispositions may also influence patients’ preferences for or against certain treatments. Table 2 provides a scenario combining the second and third type of influence and its ethical analysis.

**Table 1:** Scenario 3: Influence of response shift on medical decision making (micro level)

Scenario 3	Ethical analysis: nonmaleficence, beneficence, autonomy
An oncologist discusses published PRO data from an RCT (see Scenario 1) with a patient with metastatic gastric cancer. Based on the PRO data, the patient prefers regimen B (the triplet of cytotoxic agents) over regimen A, because QoL scores of this group are higher at follow-up. Whether or not the patient would undergo the same response shift as the study respondents is not certain. Not knowing about the response shift causing the higher HRQoL scores means that the patient’s decision is not fully informed. Consequently, the patient may be overtreated, resulting in unnecessary side-effects and lower health state at follow-up than regimen A would have yielded.	The example is problematic from the perspective of nonmaleficence. At the moment of the decision, no harm is done yet. However, the overtreatment that may be the consequence, leading to a worse health state, equals ‘doing harm’. In addition, the principle of autonomy is at stake as well, since the decision is not fully informed. Whereas possible differences between study groups and the individual patient - such as gender, age, and possibly lifestyle - are ideally taken into consideration, influences of response shifts and dispositions are less well-known and rarely discussed in SDM. However, the patient is still included in the decision making and informed about options, expected benefits and risks. Therefore, this may be considered only a minor violation of the autonomy principle, especially as it is not possible to tease out all health changes from response shift and disposition in PRO data.

**Table 2:** Scenario 4: Influence of disposition on medical decision making (micro level)

Scenario 4	Ethical analysis: nonmaleficence, beneficence, autonomy
A cardiologist sees a patient with stable coronary artery disease and low ischemic burden, and consequently no indication for coronary angioplasty. The patient reports four occurrences of chest pain per day. Due to high trait anxiety, he is not only vulnerable to over-perceiving heart symptoms, but also inclined to catastrophize the occurrences of chest pain. [13] Furthermore, his anxiety about the chest pain results in a strong preference for angioplasty over continuing conservative treatment (medication). Finally, the cardiologist decides to refer the patient for angioplasty, leading to medically unnecessary treatment [14] and consequently unnecessary medical risks.	The disposition of the cardiac patient influences his self-evaluation as well as his treatment preference (requesting angioplasty). The consequent unnecessary treatment is in conflict with both the beneficence and nonmaleficence principles. As there are no health benefits that outweigh the health risks of the intervention, the treatment is not in the best interest of the patient and the health risks imply possible harm. Whereas the treatment may comfort this anxious patient, leading to a (presumably temporary) improvement in self-reported health or wellbeing, it would have been better to refer the patient for treatment of his anxiety. Concerning the principle of autonomy, the situation does not seem problematic as it is the patient’s own self-evaluation and preference that informs the decision leading to sub-optimal care. However, the patient is probably unaware of the influence of disposition on his self-evaluation. Not being able to take this into account raises the question of whether the decision is optimally informed and, consequently, autonomous.

### Decisions in healthcare policy: ethical theories of distribution

On the macro level, PRO data from clinical studies are used for decisions in healthcare policy. Below we discuss different types of decisions that may be influenced by response shift and disposition, and analyse examples from an ethical perspective.

#### Two ethical theories of distribution

We use two of the ethical theories of distribution that are relevant for the macro level and frequently guide health policy decisions in Western European countries: classical utilitarianism and fair equality of opportunity.

Classical utilitarianism is a consequentialist theory usually associated with the work of the philosophers Jeremy Bentham and John Stuart Mill. It states that actions are just when they maximize ‘utility’, usually defined as wellbeing, welfare, or happiness. According to Mill’s account of ‘hedonistic’ utilitarianism, decisions should lead to the greatest happiness for the greatest number of people, i.e. maximizing pleasure and minimizing pain.

When applied to healthcare, utilitarianism implies maximizing total (expected) utility within the boundaries of limited healthcare resources, regardless of how resources and utility are distributed [15]. People may differ in how much utility they can ‘derive’ from the same amount of resources (‘capacity to benefit’). For example, one patient might benefit more from a certain treatment than another patient, in terms of health or wellbeing [16]. Following health economics, we take peoples’ valuations of their health-related quality of life as the ‘good’ that should be maximized, indicating the relative desirability of these health states. The utility of a medical treatment is thus the valuation of the incremental quality of life, combined with the duration of the quality-of-life levels.

‘Fair equality of opportunity’ is the egalitarian account of Norman Daniels, applying Rawls’ ‘Theory of justice’ to healthcare. It considers the protection of the ability of individuals to participate in the political, social, and economic life of their society [17]. According to Daniels, by keeping people close to ‘normal functioning’, healthcare can provide people their fair share of the ‘societal normal range of opportunities’ that reasonable people would choose in that society. Applied to decisions in healthcare policy, it is this functioning that is taken into account and not the impact of disease and treatment on patients’ wellbeing, happiness, or other types of utility [18]. Thus, fair equality of opportunity implies that every patient should have access to a certain minimum level of healthcare, to promote normal functioning and thus protect fair equality of opportunity [19]. This also implies that people with severe illness or disabilities who nevertheless report high levels of life satisfaction or quality of life can still appeal to support in obtaining a fair share of an opportunity range, because they have an objective loss in their range of capabilities and opportunities [20].

### Healthcare policy decisions

We distinguish between two types of healthcare policy decisions that may be affected by response shifts and dispositions. The first type is devising treatment guidelines for specific conditions, to designate which treatment is preferred. For some conditions, these decisions are based on data from RCTs (see Table 3).

**Table 3:** Scenario 5: Influence of response shift on guidelines (macro level)

Scenario 5	Ethical analysis: Utilitarianism	Ethical analysis: Fair equality of opportunity
An RCT is conducted to compare the effects of bypass surgery (open heart surgery) and angioplasty (catheter intervention) on frail patients. In the longer term, both treatments produced the same health status. However, as bypass surgery requires several months of recovery and thus adaptation, it may induce a greater response shift than angioplasty. As a result, after 6 months the bypass group reports higher levels of HRQoL than the angioplasty group, even though their health states are similar. This shows that the guidelines may be suboptimal, with an unwarranted preference for bypass surgery, leading to suboptimal care: unneeded treatment with unnecessary medical risks.	Since utility should be maximized, influences of response shifts or dispositions on self-evaluations are not an issue as such. The situation is problematic because bypass surgery is more expensive than angioplasty and has more medical risks, in this case without greater health benefits. However, the higher HRQoL scores due to response shift may justify the preference for bypass surgery, despite the medical risks. Nonetheless, especially when the costs and risks of bypass surgery are substantially higher, one might question whether these ‘extra’ resources would not be better spent on other healthcare or even services other than healthcare. Indeed, this may yield a larger increase of total utility in the broad sense, i.e. the wellbeing of the population.	The situation is problematic. The guideline may lead to medical risks of unneeded bypass surgery, which could cause a loss in the range of capabilities and opportunities of this patient group.

In the second type, PRO data are used in cost-effectiveness analyses to decide which treatments should be included or excluded in the basic healthcare package. Response shift and disposition may influence these decisions at two points. First, as explained above, response shifts may influence PROs, e.g. EuroQol (EQ-5D) health questionnaire data. Second, such PRO data are combined with ‘utility tariffs’, to calculate the utility of a treatment in terms of quality-adjusted life years (QALYs). Utility tariffs are valuations of health states, indicating the relative desirability of these health states. Utility is anchored at 0 (as bad as death) and 1 (as good as perfect health). For reasons of democratic legitimacy, most national guidelines require that utility tariffs are estimated from the public’s perspective. These tariffs thus reflect how the general public *values* health states as *described* by patients. Generally, valuations by the general public are lower than patient valuations, which may be affected by response shifts induced by disease experience – one of the known causes of this discrepancy [21]. However, the size of the discrepancy between valuations from the public and patients may vary, depending on health states and patient groups. For example, there are indications that larger discrepancies may be expected for patients with worse health states [22]. As a result, the cost-effectiveness analyses may lead to different conclusions than if the utility scores of patients had been used (see Table 4).

**Table 4:** Scenario 6: Influence of response shift on inclusion in healthcare package (macro level)

Scenario 6	Ethical analysis: Utilitarianism	Ethical analysis: Fair equality of opportunity
A cost-effectiveness (costs per QALY) study is carried out among patients with Crohn’s disease. Treatment A (standard care) is a colostomy, after which patients need to use stoma bags. Treatment B delays the need for a colostomy for 6 years, has no side-effects, and costs EUR 53,000. The total costs of stoma care for 6 years (group A) are estimated at EUR 7000. Treatment B thus costs EUR 46,000 more than standard care. Utility is determined from the perspective of the general public. Based on a scenario describing aspects of life with a stoma, the general public estimates life with a stoma at a value of 0.8. [23] Treatment B would increase this utility from 0.8 to 1.0 for 6 years. Treatment B would thus yield 0.2 × 6 = 1.2 QALY, at incremental costs of EUR 46,000. Thus, treatment B has a cost-effectiveness of EUR 38,000 per QALY, which is acceptable in most Western countries. However, the health valuations by patients with colostomies are significantly higher, at 0.92, [23] probably partly due to response shift. If the patients’ own utility scores had been used, the incremental utility would only be 0.08 for 6 years, leading to a smaller incremental value of 0.08 × 6 = 0.48 QALYs. Combined with the incremental costs of EUR 46,000, the cost-effectiveness would be EUR 96,000 per QALY, which might not be acceptable in many Western countries. [24] Thus, using the valuations of the general public, treatment B would be reimbursed, while it would not if patients were asked to value their own health states.	The situation is not problematic. Using utility tariffs derived from the general public instead of the patient group for cost-effectiveness analyses does not conflict with a utilitarian point of view. Utilitarianism does include the option to let society determine the desirability or undesirability of health states. In other words, it may be left to the general public to determine how ‘bad’ it considers certain health states to be, and the amount of money it is willing to spend to improve these health states.	The situation is problematic in the sense that only health benefits that improve *functioning* should be taken into account in decisions for reimbursement, instead of self-reported HRQoL (including influences of response shift and disposition) or valuations of health states (utility). Thus, using utility tariffs is always in conflict with the theory of fair equality of opportunity. The more utility tariffs (derived from the general public) differ from the actual health states of patients, the more problematic it becomes. In this case, the patients’ valuations would be higher than the valuations derived from the general public, partly due to response shift. Therefore, using the valuations of the general public is less problematic than using patient valuations. In this particular example, using valuations of the general public leads to reimbursement of treatment B, with six extra years of functioning without having to use stoma bags. Thus, the patients’ range of capabilities and opportunities is optimally protected.

## Conclusions

Response shifts tend to mitigate or amplify changes in PROs, and differences in disposition may lead to different PRO scores among people with the same health state. This may influence medical decisions at both the levels of individual patients and health policy, leading to suboptimal care.

The question arises of how serious the consequences are if these influences are not considered. The answer is not only dependent on empirical data and the ethical theory applied but also on the health concept used. As Haverkamp et al. have shown, different practices may require different concepts of health [25]. In this paper, we have used the biomedical concept of health. Other, broader conceptions of health have been proposed and debated, including positively phrased definitions of health such as ‘overall physical, mental and social wellbeing’ [26] and ‘the ability to adapt and self manage’ [27]. From these perspectives, healthcare should aim to improve biological functioning as well as to improve overall wellbeing and adaptation. Thus, in these latter approaches, the influences of response shifts and dispositions on PROs may be viewed as beneficial. However, as with the biomedical perspective, these influences still need to be teased apart from actual health states. We believe that patients may be entitled to know about the influences of response shifts and dispositions on PROs that inform their treatment decisions. Healthcare practitioners may need to learn about their patients’ dispositions and how patients adapt to their disease in order to provide good care. Moreover, patients who have adapted to symptoms and functional problems or who are not inclined to report them may still benefit from treating these burdens of disease.

Also on the level of healthcare policy, medical decision making could benefit from taking into account the influence of response shift and disposition. The ethical analysis of the scenarios presented above also show that decisions about guidelines and reimbursement of treatments may not be fully informed. Not only reflecting on the possible influences of response shift could enhance the decision making; the different ethical perspectives and conceptions of health and their differential implications for healthcare policy also need to be considered.

Pertinent questions arise from a biomedical perspective towards health. For example, how many decisions are influenced by response shift and disposition, and result in sub-optimal care, health inequities, or inefficient use of healthcare resources? Does it make a difference ethically if under- or over-treatment is caused by the influences of response shifts or dispositions? What is more problematic: unnecessary treatments for demanding patients, or under-treating patients who downplay their symptoms?

Given the importance of the patient’s perspective in healthcare and research, and the fact that PROs cannot be replaced by clinical measures, it is our intention to improve rather than criticize the use of PROs. Our aim is to raise awareness of the potential influences of disposition and response shifts on medical decisions via PROs. We call on healthcare practitioners, researchers, policy makers, health insurers, and other stakeholders to critically reflect on how and why such patient reports are used. For example, is the aim to assess the impact of a treatment on patients’ wellbeing, or on their health state? We would particularly encourage healthcare practitioners to ask patients more probing questions about symptoms and functional problems, or how they respond to a certain treatment. Existing SDM training programmes for healthcare practitioners could incorporate the subjects of response shift and dispositions to provide them with the knowledge and skills needed to explain such influences to their patients. It is also our hope that this reflection will stimulate empirical research into the effects of response shift and dispositions on medical decision making. In cross-sectional and prospective studies, dispositions could be assessed to investigate their influence on PROs and possibly enable the correction of these influences in future research. Considering response shift, as a first step, we need to investigate which types of treatments are likely to induce response shifts. Knowledge about the PROs that are most susceptible to response shifts [28] and statistical techniques distinguishing response shifts from actual health changes are available [29]. We thus have the tools to start the investigation, with the aim of improving the use of PROs in medical decision making.

## Data Availability

Not applicable.

## Abbreviations

HRQoL: health-related quality of life
PRO: patient-reported outcome
QALY: quality-adjusted life year
RCT: randomized controlled trial
SDM: shared decision making
